# Altered autonomic cardiovascular function in adults with persisting post‐concussive symptoms and exercise intolerance

**DOI:** 10.14814/phy2.70378

**Published:** 2025-06-11

**Authors:** Leah J. Mercier, Samantha J. McIntosh, Joel S. Burma, Julia Batycky, Julie M. Joyce, Jean‐Michel Galarneau, Michael J. Esser, Kathryn J. Schneider, Jonathan D. Smirl, Sean P. Dukelow, Ashley D. Harris, Chantel T. Debert

**Affiliations:** ^1^ Department of Clinical Neurosciences, Division of Physical Medicine and Rehabilitation University of Calgary Calgary Alberta Canada; ^2^ Hotchkiss Brain Institute (HBI), University of Calgary Calgary Alberta Canada; ^3^ Sport Injury Prevention Research Centre (SIPRC), Faculty of Kinesiology University of Calgary Calgary Alberta Canada; ^4^ Human Performance Laboratory, Faculty of Kinesiology University of Calgary Calgary Alberta Canada; ^5^ Libin Cardiovascular Institute of Alberta University of Calgary Calgary Alberta Canada; ^6^ Alberta Children's Hospital Research Institute (ACHRI) University of Calgary Calgary Alberta Canada; ^7^ Cerebrovascular Concussion Laboratory, Faculty of Kinesiology University of Calgary Calgary Alberta Canada; ^8^ Department of Radiology University of Calgary Calgary Alberta Canada; ^9^ Department of Pediatrics, Section of Neurology University of Calgary Calgary Alberta Canada; ^10^ Sports Medicine Centre University of Calgary Calgary Alberta Canada

**Keywords:** baroreceptor, blood pressure, concussion, heart rate variability, mild traumatic brain injury, persisting post‐concussive symptoms

## Abstract

Alterations in autonomic cardiovascular function may result following mild traumatic brain injury (mTBI), but there is a lack of data evaluating autonomic function in adults with persisting post‐concussive symptoms (PPCS). We collected resting measures of heart rate (HR), heart rate variability (HRV), blood pressure (BP) and cardiac baroreceptor sensitivity in 50 adults with PPCS (42.8 (11.0) years; 24.5 (14.2) months post‐injury; 74% female) and 50 age/sex‐matched controls (43.0 (11.1) years; 74% female) with no lifetime mTBI history. HR and BP data were collected for ≥4 min when seated and standing. Between‐group differences (PPCS vs. control) were analyzed using linear regression. In the seated posture, participants with PPCS had significantly lower HRV than controls, specifically RMSSD (root mean squared of successive RR interval differences) (mean difference = −10.450, *p* = 0.001) and SDNN (standard deviation of RR intervals) (mean difference = −12.875, *p* = 0.001). In the standing position, PPCS had lower SDNN. Participants with PPCS had significantly lesser change in HRV, including RMSSD (mean difference = 5.981, *p* = 0.007) and LF/HF (low‐frequency to high‐frequency ratio) (mean difference = −2.229, *p* = 0.016), when going from seated to standing compared to controls. Findings suggest adults with PPCS have altered autonomic function relative to controls. Physical activity level and deconditioning are potential treatment targets to improve autonomic function.

## 1 INTRODUCTION

The average length of recovery from mild traumatic brain injury (mTBI) may depend on the mechanism of injury and the studied patient population; however, a significant proportion, up to 31%, of individuals who sustain an mTBI go on to have persisting post‐concussive symptoms (PPCS) beyond 1 month (Cancelliere et al., 2023; McMahon et al., 2014; Patricios et al., 2023). PPCS presentation varies between individuals, but commonly includes headaches, dizziness, fatigue, cognitive difficulties, low mood, and exercise intolerance (Antonellis et al., 2024; Cancelliere et al., 2023; Kozlowski et al., 2013). Exercise intolerance is characterized by the exacerbation and/or reappearance of post‐concussive symptoms during exercise (Antonellis et al., 2024; Galea et al., 2022). Symptom exacerbation generally worsens as heart rate (HR) increases (Clausen et al., 2016; Pelo et al., 2023). The underlying pathophysiology of exercise intolerance is poorly understood but may be related to altered cerebral blood flow response to exercise and/or autonomic cardiovascular dysfunction resultant either from the initial injury or a period of deconditioning following mTBI. Exercise intolerance, among other factors (e.g., kinesiophobia, fear avoidance, prescription of prolonged rest, post‐concussive symptom burden), likely contributes to physical inactivity in adults with PPCS and absence of return to pre‐morbid activity level (Mercier et al., 2021). To date, most research evaluating autonomic function following mTBI has recruited athletic cohorts in the sub‐acute phase (within 1–2 weeks) following injury or asymptomatic cohorts in the chronic phase (>1–3 months) of injury (Mercier et al., 2022; Pelo et al., 2023). While there is evidence for improvement in autonomic cardiovascular function over time following injury (Purkayastha et al., 2019), it is not known whether differences in autonomic parameters exist in adults with months‐to‐years of PPCS compared to matched controls. This study adds to the existing literature by evaluating autonomic function in a sample of adults with all‐cause mTBI (i.e., motor vehicle collision, falls, sport‐related). As the majority of work in this area has been conducted in adolescent sport‐related concussion, additional studies in adults with diverse mechanisms of injury are needed to determine the generalizability of initial findings describing post‐traumatic alterations in autonomic function.

Evaluation of heart rate variability (HRV) and cardiac baroreceptor sensitivity (BRS) are two commonly used metrics to evaluate autonomic cardiovascular function in the field of concussion (Mercier et al., 2022). These metrics can be monitored non‐invasively in a variety of test conditions (rest, exercise, physiologic challenge) using ultra‐short term (<3 min), short‐term (~5 min), or long‐term (24 h) recordings (Mercier et al., 2022; Shaffer & Ginsberg, 2017). HRV is the variation in time interval between consecutive heartbeats (Nunan et al., 2010; Shaffer & Ginsberg, 2017). These oscillations in HR allow for rapid adaptation of the cardiovascular system to homeostatic challenge and present an important measure of cardiac health (Nunan et al., 2010; Shaffer & Ginsberg, 2017). HRV can be characterized by time‐domain, frequency‐domain, and/or non‐linear indices (Nunan et al., 2010; Shaffer & Ginsberg, 2017). Time‐domain measures quantify the amount of variability between interbeat intervals. Commonly reported time‐domain metrics include SDNN (standard deviation of RR intervals), RMSSD (root mean squared of successive RR intervals) and pNN50 (percentage of successive RR intervals differing by >50 ms) (Shaffer & Ginsberg, 2017). Frequency‐domain metrics serve as proxies for the relative contributions of the sympathetic and/or parasympathetic nervous system by reporting on the power of different frequency bands (Shaffer & Ginsberg, 2017). High‐frequency (HF) fluctuations, often called the “respiratory band”, are frequencies of 0.15–0.4 Hz, largely embodying parasympathetic outflow (Shaffer & Ginsberg, 2017). Low‐frequency (LF) fluctuations are frequencies of 0.04–0.15 Hz and are interpreted to reflect both sympathetic and parasympathetic nervous system outflow (Shaffer & Ginsberg, 2017). Non‐linear parameters (including SD1 and SD2) are often conceptualized as measures of HR unpredictability or complexity (Piskorski & Guzik, 2021; Shaffer & Ginsberg, 2017). Finally, cardiac BRS has been employed to index the function of the carotid and aortic baroreceptors to modulate HR and blood vessel diameter based upon brief changes in BP (La Rovere et al., 2008). The most widely used metric to quantify this response is LF gain, measured as the ratio between change in HR and blood pressure (BP) (Burma et al., 2020; Smirl et al., 2022).

There is increasing evidence showing altered autonomic cardiovascular function within 72 h of injury and up to years post‐mTBI (Mercier et al., 2022; Pelo et al., 2023). Metrics of autonomic function (LF, HF, SDNN) have been associated with clinical symptom burden (Brandt et al., 2021; Sung, Chen, et al., 2016; Sung, Lee, et al., 2016) and have been shown to improve over time (with recovery) in some cases (Purkayastha et al., 2019). However, the clinical implications of these findings for diagnosis or monitoring recovery remain unclear. To date, autonomic cardiovascular function (specifically, HRV, BP, and BRS) in adults with months‐to‐years of PPCS and exercise intolerance has not been well characterized. Therefore, this study aimed to: (1) characterize resting HR, HRV, BP, and BRS in seated and standing postures; and (2) evaluate change in autonomic metrics from seated to standing in participants with PPCS compared to age/sex‐matched controls. We hypothesized participants with PPCS would have significantly higher resting seated and standing HR and BP and significantly lower time‐domain HRV relative to age/sex‐matched controls.

## 2 MATERIALS AND METHODS

### 2.1 Study design

This cross‐sectional study was nested within a larger randomized controlled trial, the ACTBI (Aerobic exercise for treatment of Chronic symptoms following mild Traumatic Brain Injury) trial (Mercier et al., 2020). The trial was registered on clinicaltrials.gov (NCT03895450). A sample size calculation was completed for the parent study based on the primary trial outcome (Mercier et al., 2020).

### 2.2 Study participants

Participants were recruited from May 2019 to February 2023. Recruitment was paused between March 2020 and June 2021 due to the COVID‐19 pandemic. All participants were screened by telephone prior to the initial assessment to discuss participation and assess eligibility. Participants provided written consent prior to the initial assessment.

Fifty‐two participants with PPCS were recruited from outpatient brain injury, chronic pain, and physiotherapy clinics in Calgary, Alberta, Canada for the parent trial. Inclusion criteria were as follows: (1) aged 18–65 years; (2) diagnosis of mTBI based on the ACRM criteria (American Congress of Rehabilitation Medicine, 1993); (3) diagnosis of PPCS based on the ICD‐10 post‐concussional syndrome criteria (World Health Organization, 1993) who were ≥3 months to 5 years post‐injury; (4) presented with exercise intolerance (acute exacerbation of post‐concussive symptoms with exercise), which was a criterion for the parent exercise intervention trial (Mercier et al., 2020); (5) stable pharmacologic regimen for ≥1 month prior to participation. Participants with PPCS were excluded if they had a history of moderate‐to‐severe TBI, neurologic, cardiopulmonary, respiratory (including persistent respiratory symptoms following COVID‐19 infection), psychiatric disorder (other than depression, anxiety and/or post‐traumatic stress disorder) or chronic musculoskeletal condition limiting engagement in exercise (criterion for parent trial (Mercier et al., 2020)).

Controls were recruited via convenience based on PPCS participant age (±3 years) and sex‐matching, with snowball sampling from the community. Exclusion criteria were: (1) any lifetime history of diagnosed or suspected traumatic brain injury; and (2) diagnosis of neurologic, cardiopulmonary, respiratory (including persistent respiratory symptoms following COVID‐19 infection) or psychiatric disorder (other than depression, anxiety and/or post‐traumatic stress disorder). Participants provided written consent prior to the assessment. All participants completed a single assessment.

### 2.3 Clinical outcome measures

All participants provided self‐reported demographic information. Participants with PPCS also provided self‐reported injury characteristics. All participants were asked to retrospectively self‐report the frequency and duration of mild, moderate, and vigorous activity completed in the week prior to assessment using a modified Godin Leisure‐Time Physical Exercise Questionnaire (GLTEQ) (Godin & Shephard, 1985). Total minutes of moderate and vigorous activity were summed to determine if physical activity guidelines (≥150 minutes/week) were met (Ross et al., 2020). Sedentariness was assessed using the Rapid Assessment Disuse Index (RADI) (Shuval et al., 2014). Participants with PPCS also completed the Rivermead Post Concussion Symptoms Questionnaire (RPQ) (King et al., 1995), a measure of symptom burden. All data were collected immediately prior to or following the study visit via completion of digital forms. For participants with PPCS, exercise intolerance was assessed using the Buffalo Concussion Treadmill Test (BCTT), a graded treadmill test used to determine HR at the point of symptom exacerbation (Leddy & Willer, 2013; Mercier et al., 2020).

### 2.4 Physiologic monitoring

All participants completed physiological monitoring under the supervision of a certified exercise physiologist. Height and weight measurements were completed to calculate body mass index (BMI). Note, for 65 participants (PPCS, *n* = 32; controls, *n* = 33), mandatory masking in the laboratory was required due to the COVID pandemic. However, participants did not wear a mask for ~5 min prior to or during physiologic monitoring. Following a rest period to establish baseline physiology, quiet, resting seated and standing HR and BP measures were collected. Four minutes of resting data were analyzed in both the seated and standing postures; data recordings of ≥4 min have been validated for evaluating short‐term HRV and shown to sufficiently approximate longer recordings (Burma, Graver, et al., 2021). A ≥1 min adaptation period was allowed following postural change (from seated‐to‐standing) to establish baseline standing physiology. Participants and research staff were instructed to refrain from talking during the recording. All participants refrained from alcohol (Brunner et al., 2021), caffeine (Grant et al., 2023) and vigorous exercise (Burma et al., 2020) for ≥6 h prior to assessments as these are confounders known to influence HRV parameters (Ellingson, Singh, Ellingson, Dech, et al., 2022). Participants who used inhalers were advised to bring them to the assessment (in case needed) but refrain from use on the day of assessment prior to testing. All assessments were conducted during standard working hours (07:30–17:00).

A 12‐lead electrocardiogram (ECG) system (CardioSoft 6.7 ECG software, GE Medical Systems, Milwaukee WI, USA), amplifier, and acquisition software were used (PowerLab 16/35 amplifier, LabChart 8 software, ADInstruments, Colorado Springs, CO, USA) for HR monitoring at 1000 Hz (Burma, Lapointe, et al., 2021). ECG was integrated with the Vmax Encore 29 Metabolic Cart (Vyaire Medical, Mettawa IL, USA). Beat‐to‐beat BP was collected using finger photoplethysmography. The finger BP cuff was placed on the left middle finger (Finapress NOVA, Finapress Medical Systems Enschede, Netherlands or Portapres Model‐2, TNO TPD Biomedical Instrumentation Amsterdam, Netherlands) and a height correction unit was placed on the participant at the level of the heart. A manual brachial BP was taken at two time points during both seated and standing periods by a trained exercise physiologist to establish a correction factor; systolic (SBP), diastolic (DBP), and mean arterial pressure (MAP) are reported.

### 2.5 Heart rate and blood pressure analysis and processing

Using ECG lead 2 from the Vmax system, PowerLab converted the signal from analog to digital, exporting the raw ECG waveform at 1000 Hz (Burma, Lapointe, et al., 2021). PowerLab was interfaced with LabChart to collect ECG and BP waveform and output. ECG data was exported as a text file and imported into Ensemble‐R software (V1.0.43, Ensemble, Elucimed, Wellington, NZ). ECG traces were visually inspected to identify any artifacts or ectopic beats. Artifacts were replaced with interpolated RR data points using an automatic correction. Recordings with multiple ectopic beats were excluded. No pre‐sets or filters were applied.

Beat‐by‐beat BP data was time aligned with ECG data on LabChart. Based on brachial BP, a correction factor was applied to SBP and DBP beat‐to‐beat data in LabChart. Following correction, BP data was exported as a text file and imported into Ensemble‐R.

Once data were normalized/cleaned, NN intervals were used to compute HRV parameters using Ensemble‐R software. Reported HRV parameters include time domain (SDNN, RMSSD, pNN50), frequency domain (LF norm, HF norm, and LF/HF ratio) and non‐linear statistics derived from Poincaré plots (SD1, SD2). Beat‐to‐beat SBP, along with NN interval data, allowed for the analysis of cardiac BRS via the LF gain metric across the frequency band of 0.04–0.15 Hz (Burma et al., 2020; Smirl et al., 2022).

### 2.6 Statistical analysis

Descriptive statistics were used to report participant characteristics. Between‐group (PPCS vs. control) demographic characteristics were compared using the chi‐square test, independent samples *t*‐test, or Mann–Whitney *U* test where appropriate. Between‐group differences in resting seated and standing HRV and BP measures as well as delta values (change from seated‐to‐standing) were analyzed using a bootstrapped (resampling was done at both levels with 1000 repetitions) random intercept linear regression clustering on matching pairs (Rabe‐Hesketh & Skrondal, 2022).

A sensitivity analysis was completed to examine the effect of medications on outcomes. Between‐group differences in resting seated and standing HRV and BP measures were analyzed using the random intercept linear regression for the following subgroups: (a) excluding PPCS participants taking cardiovascular medications; or (b) pain/anti‐epileptic medications; or (c) anti‐depressant/anti‐psychotic/neurostimulant medications; or (d) tri‐cyclic anti‐depressants (TCAs); or (e) selective serotonin reuptake inhibitor (SSRIs). If a participant with PPCS was taking a medication in an above grouping, their matched pair (control) was also excluded from that analysis.

Results with a *p*‐value of <0.05 were considered statistically significant on an a‐priori basis. All analyses were conducted using Stata (StataCorp. 2021. Stata Statistical Software: Release 18. College Station, TX: StataCorp LLC) (StataCorp, 2023). Mean differences with 95% confidence intervals are reported.

## 3 RESULTS

**FIGURE 1:**
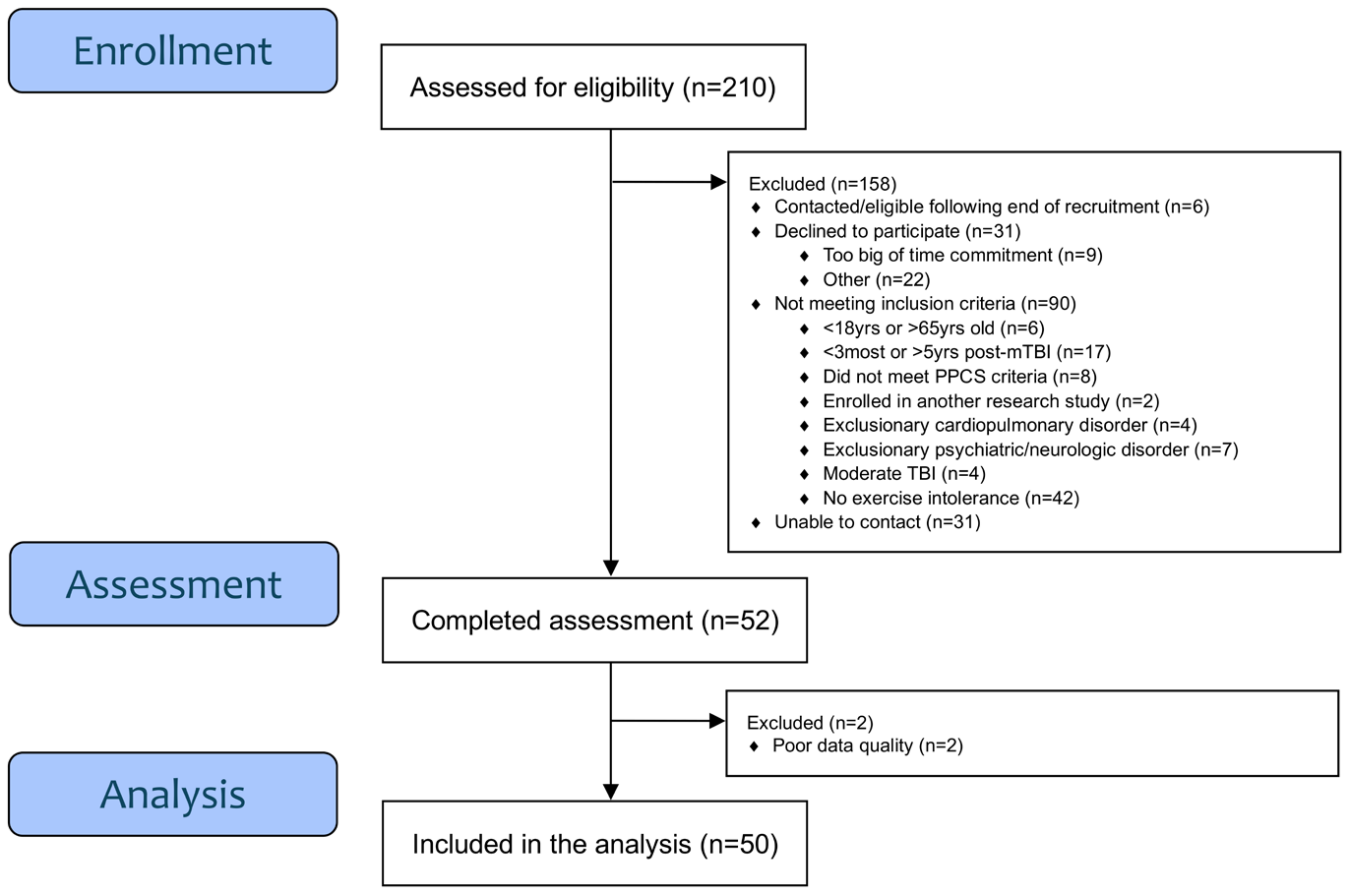
Recruitment flowchart: Participants with PPCS.

**FIGURE 2:**
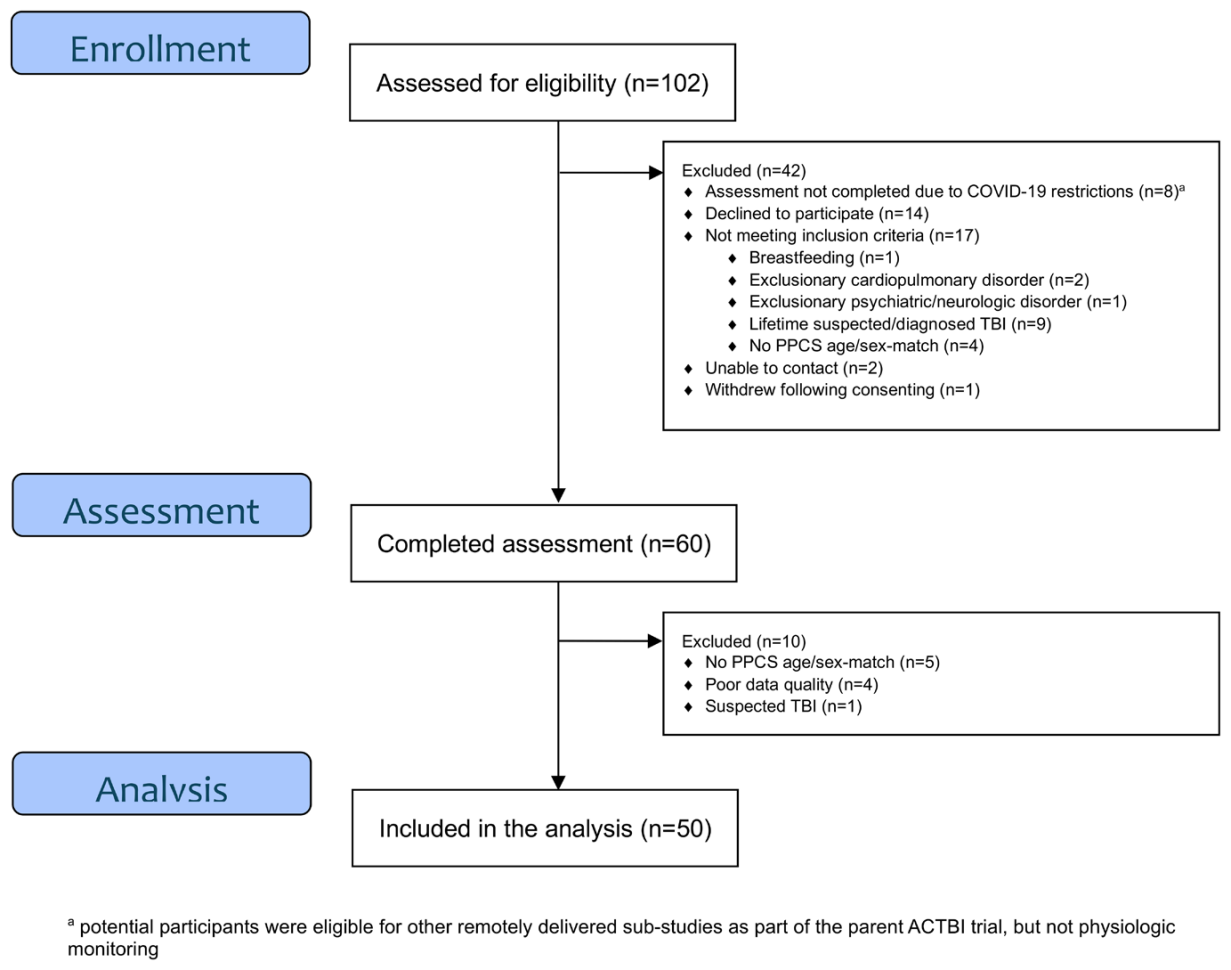
Recruitment flowchart: Age/sex‐matched controls.

Of the 210 participants with PPCS screened, 52 met inclusion criteria, were consented, and enrolled in the parent trial. Seated and standing data from two participants, one participant's seated data and one participant's standing data were excluded due to poor quality, and thus 49 participants with PPCS were included in each of the seated and standing analyses. A total of 102 control participants were screened, of which 60 completed an assessment. Data from 10 participants were excluded either due to poor quality (*n* = 4), lack of PPCS participant match (*n* = 5) or previous suspected TBI not disclosed during screening (*n* = 1). See Figures 1 and 2 for recruitment details.

### 3.1 Participant characteristics

Participants with PPCS were a mean of 42.8 (11.0) years old and 74% female. No participants had a clinical diagnosis of postural orthostatic tachycardia syndrome (POTS) or other clinical autonomic disorders. One participant with PPCS was a current tobacco smoker reporting smoking 3 packs of cigarettes per week; otherwise, no nicotine use was reported. Matched controls were a mean of 43.0 (11.1) years old (74% female), none of whom reported nicotine use. Demographic characteristics of participants with PPCS and age/sex‐matched controls are presented in Table 1.

**TABLE 1:** Participant characteristics.

	PPCS (*n* = 50)	Controls (*n* = 50)	*p* Value
Age (y), mean (SD)	42.8 (11.0)	43.0 (11.1)	0.928
Sex, *n* (% female)	37 (74.0)	37 (74.0)	>0.999 [*χ* ^2^ < 0.001]
Handedness, *n* (% right‐handed)	46 (92.0)	43 (86.0)	0.259 [*χ* ^2^ = 2.701]
Height (cm), mean (SD)	169.8 (9.0)	167.2 (9.0)	0.218
Weight (kg), mean (SD)	78.9 (23.3)	72.6 (13.7)	0.410
BMI (kg/m^2^), mean (SD)	27.2 (7.1)	25.9 (4.3)	0.978
Education, *n* (%)			
PhD/MD/JD	2 (4.0)	7 (14.0)	0.308 [*χ* ^2^ = 4.802]
Master's Degree	10 (20.0)	10 (20.0)	
Bachelor's Degree	21 (42.0)	20 (40.0)	
Trade school/vocational education	11 (22.0)	11 (22.0)	
Grade 12 or less	6 (12.0)	2 (4.0)	
Current work status, *n* (%)			
Employed	30 (60.0)	41 (82.0)	
Student	2 (4.0)	5 (10.0)	
Not currently working, but was employed/student prior to mTBI	16 (32.0)		
Not employed nor a student	2 (4.0)	4 (8.0)	
Medical history, *n* (%)			
Endocrine	9 (18.0)	3 (6.0)	
Headaches^a^	4 (8.0)	7 (14.0)	
Hypertension	4 (8.0)	0 (0.0)	
Mental health diagnosis^a^	11 (22.0)	7 (14.0)	
Sleep disorder	4 (8.0)	2 (8.0)	
Other	28 (56.0)	19 (38.0)	
Medications, *n* (%)			
Anti‐depressant/anti‐psychotic/neurostimulant	27 (54.0)	2 (4.0)	
Anti‐epileptic	4 (8.0)	0 (0.0)	
Cardiovascular	3 (6.0)	0 (0.0)	
Endocrine	7 (14.0)	3 (6.0)	
Headache (preventative/abortive)	10 (20.0)	0 (0.0)	
HRT	2 (4.0)	1 (2.0)	
Metabolic	5 (10.0)	2 (4.0)	
Pain	14 (28.0)	0 (0.0)	
Respiratory/Antihistamine	4 (8.0)	3 (6.0)	
Sleep	15 (30.0)	0 (0.0)	
Other	8 (16.0)	7 (14.0)	
Meeting PA guidelines^b^, *n* (% yes)	8 (16.0)	24 (72.7)	<0.001* [*χ* ^2^ = 27.004]
Moderate‐to‐vigorous physical activity^b^ (mins), mean [range]	72 [0–420]	378 [0–1890]	
Pre‐injury participation in sport^c^, *n* (% yes)	15 (30.0)		
BCTT stage reached, mean (SD)	8.7 (4.1)		
Baseline questionnaires, mean (SD)			
RPQ [0–64]	35.1 (9.4)		
RADI [3–15]	9.4 (2.5)	9.15 (2.2)	0.593

Abbreviations: BMI, body mass index; BCTT, Buffalo Concussion Treadmill Test; HRT, hormone replacement therapy; JD, Juris Doctor; MD, Doctor of Medicine; mTBI, mild traumatic brain injury; PhD, Doctor of Philosophy; PA, physical activity; PPCS, persisting post‐concussive symptoms; RADI, Rapid Assessment Disuse Index; RPQ, Rivermead Post Concussion Symptoms Questionnaire.

* Statistically significant at *p* < 0.05.

^a^ For PPCS participants, pre‐injury diagnosis.

^b^ Based on modified Godin Leisure‐Time Physical Activity Questionnaire; *n* = 33 controls.

^c^ Response to question, “At the time of your brain injury were you involved in organized or competitive athletics?”

Participants with PPCS were a mean of 24.5 (14.2) months post‐injury. The most common mechanisms of injury were motor vehicle collision (42%) and sport‐related injury (30%). Injury characteristics are presented in Table 2.

**TABLE 2:** PPCS injury characteristics.

	Participants with PPCS (*n* = 50)
Months since injury, mean (SD)	24.5 (14.2)
Mechanism of injury, *n* (%)	
Sports/recreation	15 (30.0)
Fall	7 (14.0)
Work‐related	2 (4.0)
MVC	21 (42.0)
Assault	3 (6.0)
Other	2 (4.0)
Loss of consciousness, *n* (%)	
Yes	13 (26.0)
No	29 (58.0)
Unknown	8 (16.0)
Post‐traumatic amnesia, *n* (%)	
Yes	21 (42.0)
No	24 (48.0)
Unknown	5 (10.0)
Lifetime mTBI, *n* (%)	
1	20 (40.0)
≥2	30 (60.0)

Abbreviations: mTBI, mild traumatic brain injury; MVC, motor vehicle collision.

Participants with PPCS reached a mean of 8.7 (4.1) stages on the BCTT, indicating a high degree of exercise intolerance. No participants required inhaler use during testing. Based on the GODIN, only 16% of participants with PPCS reported meeting physical activity guidelines. In response to the question, “Compared to prior to your most recent brain injury, to what degree have you returned to your previous level of exercise?” (0% = absence of any exercise; 100% = return to the same level/amount of exercise as prior to injury), participants with PPCS reported having returned to a mean of 28% of pre‐morbid activity. Only 22% of participants reported having returned to ≥50% of pre‐morbid activity.

Medications taken by PPCS participants are reported in Table S1. Concurrent therapies that PPCS participants were engaged in are reported elsewhere (Mercier et al., 2025).

### 3.2 Resting HRV and BP

Results of models evaluating HRV and BP outcomes between groups (PPCS vs. age/sex‐matched controls) are presented in Table 3. Multiple HRV and BP parameters significantly differed between the PPCS and control groups during seated rest. Models revealed significantly higher HR (mean difference = 5.381, 95% CI [1.787, 8.976], *p* = 0.003), SBP (mean difference = 4.694, 95% CI [0.242, 9.146], *p* = 0.039), DBP (mean difference = 5.469, 95% CI [2.355, 8.584], *p* = 0.001) and MAP (mean difference = 5.211, 95% CI [2.114, 8.308], *p* = 0.001) in the PPCS group compared to controls. Several time domain HRV measures were significantly lower in the PPCS group, including pNN50 (mean difference = −8.524, 95% CI [−14.088, −2.959], *p* = 0.003), RMSSD (mean difference = −10.450, 95% CI [−16.828, −4.072], *p* = 0.001), and SDNN (mean difference = −12.875, 95% CI [−20.347, −5.402], *p* = 0.001). SD1 (mean difference = −7.404, 95% CI [−11.997, −2.812], *p* = 0.002) and SD2 (mean difference = −16.703, 95% CI [−27.282, −6.124], *p* = 0.002) were also significantly lower in participants with PPCS compared to controls at seated rest.

**TABLE 3:** Resting seated and standing HRV and BP in PPCS vs. age/sex‐matched controls.

	Seated comparison					Standing comparison				
	PPCS (*n* = 49)	Controls (*n* = 49)	Mean difference	95% CI	*p* Value	PPCS (*n* = 49)	Controls (*n* = 49)	Mean difference	95% CI	*p* Value
	Mean (SE)	Mean (SE)				Mean (SE)	Mean (SE)			
HR (bpm)	74.3 (1.5)	68.9 (1.2)	5.381	(1.787, 8.976)	0.003*	81.7 (1.7)	79.3 (1.6)	2.463	(−1.964, 6.891)	0.275
pNN50 (%)	9.0 (2.0)	17.5 (2.1)	−8.524	(−14.088, −2.959)	0.003*	4.2 (1.2)	4.9 (1.0)	−0.638	(−3.673, 2.397)	0.680
RMSSD (ms)	27.1 (2.7)	37.6 (2.5)	−10.450	(−16.828, −4.072)	0.001*	18.9 (1.9)	23.0 (1.6)	−4.070	(−8.822, 0.682)	0.093
SDNN (ms)	44.6 (3.4)	57.4 (2.8)	−12.875	(−20.347, −5.402)	0.001*	37.8 (2.8)	46.5 (2.4)	−8.698	(−15.496, −1.901)	0.012*
LF norm (n.u.)	68.7 (2.5)	63.6 (2.5)	5.120	(−1.617, 11.858)	0.136	77.0 (1.9)	78.4 (1.9)	−1.413	(−7.028, 4.202)	0.622
HF norm (n.u.)	27.3 (2.3)	33.0 (2.4)	−5.723	(−11.968, 0.521)	0.072	19.6 (1.6)	18.5 (1.7)	1.047	(−3.809, 5.902)	0.673
LF/HF	4.5 (0.7)	3.2 (0.5)	1.272	(−0.136, 2.680)	0.077	6.2 (0.7)	7.0 (0.9)	−0.739	(−2.960, 1.482)	0.514
SD1 (ms)	19.2 (1.9)	26.6 (1.8)	−7.404	(−11.997, −2.812)	0.002*	13.4 (1.4)	16.3 (1.2)	−2.882	(−6.388, 0.623)	0.107
SD2 (ms)	59.7 (4.7)	76.4 (3.7)	−16.703	(−27.282, −6.124)	0.002*	51.6 (3.8)	63.4 (3.2)	−11.748	(−21.562, −1.935)	0.019*
SBP^a^ (mmHg)	115.1 (1.8)	110.4 (1.5)	4.694	(0.242, 9.146)	0.039*	111.9 (1.8)	110.5 (1.7)	1.408	(−3.048, 5.864)	0.536
DBP^a^ (mmHg)	75.5 (1.2)	70.0 (1.0)	5.469	(2.355, 8.584)	0.001*	75.2 (1.2)	72.4 (1.3)	2.806	(−0.427, 6.039)	0.089
MAP^a^ (mmHg)	88.7 (1.3)	83.5 (1.1)	5.211	(2.114, 8.308)	0.001*	87.4 (1.2)	85.1 (1.3)	2.340	(−0.799, 5.480)	0.144
LF gain^b^ (ms/mmHg)	9.9 (1.0)	12.2 (1.1)	−2.329	(−5.249, 0.591)	0.118	7.2 (0.6)	8.7 (0.8)	−1.438	(−3.024, 0.148)	0.076

*Note*: Mean difference and 95% confidence intervals from the random intercept model of resting seated and standing HRV and BP between participants with PPCS and controls.

Abbreviations: BP, blood pressure; DBP, diastolic blood pressure; HR, heart rate; HRV, heart rate variability; HF norm, high frequency power in normalized units; LF gain, low frequency gain; LF norm, low frequency power in normalized units; LF/HF ratio, low frequency to high frequency ratio; MAP, mean arterial pressure; pNN50, percent of RR intervals differing by more than 50 ms; PPCS, persistent post‐concussive symptoms; RMSSD, root mean squared of successive RR interval differences; SBP, systolic blood pressure; SDNN, standard deviation of RR intervals; SD1, Pointcaré plot standard deviation perpendicular to the line of identity; SD2, Pointcaré plot standard deviation along the line of identity.

* Statistically significant at *p* < 0.05.

^a^ Average of two manual brachial BPs.

^b^ *n* = 27 PPCS; *n* = 27 control.

Models evaluating differences in HRV and BP outcomes in the standing posture revealed SDNN (mean difference = −8.698, 95% CI [−15.496, −1.901], *p* = 0.012) and SD2 (mean difference = −11.748, 95% CI [−21.562, −1.935], *p* = 0.019) were significantly lower in those with PPCS compared to controls. During standing, there were no significant differences in BP between groups.

Cardiac BRS (LF gain) did not significantly differ between groups in the seated or standing posture. Outcomes in the seated and standing postures for each group are presented in Table 3 and Figure S1.

### 3.3 Resting HRV and BP – Sensitivity analysis

Result of models evaluating HRV and BP outcomes between groups (PPCS vs. age/sex‐matched controls) for all participants except those taking cardiovascular medications or pain/epileptic medications is presented in Tables S2 and S3. Results largely mirror those of the primary analysis.

Results of models evaluating HRV and BP outcomes between groups for all participants except those taking anti‐depressants or anti‐psychotic or neurostimulant medications are presented in Table S4. Seated (*n* = 22) and standing (*n* = 23) HR, HRV, and BP outcomes were no longer significant when analyzing this smaller sub‐group of participants.

Results of models evaluating HRV and BP outcomes between groups for all participants except those taking TCA or SSRI medications is presented in Tables S5 and S6. Results largely confirmed findings of the primary analysis.

### 3.4 Difference between resting seated and standing HRV and BP

Delta values were calculated (standing value – seated value) to examine the change in values from the seated‐to‐standing posture for each group (PPCS vs. controls). Results are presented in Table 4. The control group had a significantly greater increase in HR (mean difference = −2.414, 95% CI [−4.116, −0.712], *p* = 0.005), LF/HF (mean difference = −2.229, 95% CI [−4.038, −0.420], *p* = 0.016), LF norm (mean difference = −7.515, 95% CI [−13.363, −1.668], *p* = 0.012), DBP (mean difference = −2.498, 95% [−4.386, −0.609], *p* = 0.010) and MAP (mean difference = −2.545, 95% CI [−4.418, −0.672], *p* = 0.008) from seated‐to‐standing. The control group had a significantly greater decrease in pNN50 (mean difference = 7.532, 95% CI [3.345, 11.720], *p* < 0.001), RMSSD (mean difference = 5.981, 95% CI [1.624, 10.339], *p* = 0.007), HF norm (mean difference = 7.432, 95% CI [2.048, 12.815], *p* = 0.007) and SD1 (mean difference = 4.240, 95% CI [1.051, 7.428], *p* = 0.009) compared to PPCS. Overall, the control group had more change in measures of HRV and BP between postures (seated‐to‐standing) compared to PPCS (see Figure S1).

**TABLE 4:** Difference between resting seated and standing HRV and BP values: PPCS versus age/sex‐matched controls.

	Delta value		Mean difference	95% CI	*p* Value
	PPCS (*n* = 48)	Controls (*n* = 48)			
	Mean (SE)				
HR (bpm)	7.8 (0.7)	10.2 (0.7)	−2.414	(−4.116, −0.712)	0.005*
pNN50 (%)	−4.9 (1.2)	−12.4 (1.7)	7.532	(3.345, 11.720)	<0.001*
RMSSD (ms)	−8.4 (1.4)	−14.4 (1.9)	5.981	(1.624, 10.339)	0.007*
SDNN (ms)	−7.3 (2.3)	−10.2 (2.0)	2.977	(−2.597, 8.551)	0.295
LF norm (n.u.)	7.2 (2.3)	14.7 (2.1)	−7.515	(−13.363, −1.668)	0.012*
HF norm (n.u.)	−6.9 (2.1)	−14.4 (1.9)	7.432	(2.048, 12.815)	0.007*
LF/HF	1.6 (0.6)	3.8 (0.7)	−2.229	(−4.038, −0.420)	0.016*
SD1 (ms)	−6.0 (1.1)	−10.2 (1.4)	4.240	(1.051, 7.428)	0.009*
SD2 (ms)	−8.8 (3.2)	−12.1 (2.7)	3.267	(−4.778, 11.311)	0.426
SBP^a^ (mmHg)	−3.0 (1.0)	−0.4 (1.0)	−2.642	(−5.325, 0.041)	0.054
DBP^a^ (mmHg)	−0.4 (0.6)	2.1 (0.7)	−2.498	(−4.386, −0.609)	0.010*
MAP^a^ (mmHg)	−1.3 (0.6)	1.3 (0.7)	−2.545	(−4.418, −0.672)	0.008*
LF gain^b^ (ms/mmHg)	−2.6 (0.8)	−3.5 (0.9)	0.891	(−1.397, 3.180)	0.445

*Note*: Delta values calculated as resting standing minus resting seated values for each outcome. Mean difference and 95% confidence intervals from models of delta values between groups.

Abbreviations: BP, blood pressure; DBP, diastolic blood pressure; HR, heart rate; HRV, heart rate variability; HF norm, high frequency power in normalized units; LF gain, low frequency gain; LF norm, low frequency power in normalized units; LF/HF, low frequency to high frequency ratio; MAP, mean arterial pressure; pNN50, percent of RR intervals differing by more than 50 ms; PPCS, persistent post‐concussive symptoms; RMSSD, root mean squared of successive RR interval differences; SBP, systolic blood pressure; SDNN, standard deviation of RR intervals; SD1, Pointcaré plot standard deviation perpendicular to the line of identity; SD2, Pointcaré plot standard deviation along the line of identity.

* Statistically significant at *p* < 0.05.

^a^ Average of two manual brachial BPs.

^b^ *n* = 27 PPCS; *n* = 27 control.

## 4 DISCUSSION

The aims of this study were to evaluate differences in autonomic function using resting HRV and BP metrics and response to postural change (seated‐to‐standing) between adults with PPCS and exercise intolerance compared to healthy controls. In the seated posture, participants with PPCS had significantly higher HR and BP and significantly lower HRV (time‐domain and non‐linear measures) compared to the control group. While standing, SDNN and SD2 remained significantly lower in the PPCS group compared to controls. Sensitivity analyses to probe the effect of participant medications on findings revealed that several between‐group differences remained significant when excluding participants with PPCS taking cardiovascular or pain/anti‐epileptic or TCA or SSRI medications. When evaluating change in metrics between seated and standing postures, controls exhibited a significantly greater change in HR, BP, and HRV parameters (pNN50, RMSSD, LF norm, HF norm, LF/HF, SD1) between postures compared to the PPCS group. Participants with PPCS self‐reported completing significantly less physical activity than age/sex‐matched controls. These findings suggest participants with PPCS are likely deconditioned relative to controls and exhibit altered autonomic cardiovascular function as evidenced by the altered response to standing.

### 4.1 Resting HRV and BP

During seated rest, participants with PPCS had significantly higher HR and BP (SBP, DBP, MAP) than controls. Several HRV metrics were significantly lower in individuals with PPCS in the seated posture, including pNN50, RMSSD, SDNN, SD1, and SD2. In the standing posture, SDNN and SD2 remained significantly lower in participants with PPCS compared to controls. Limited data has been published evaluating autonomic function in the chronic phase of recovery in symptomatic individuals. Available data during the sub‐acute phase of mTBI recovery have produced conflicting results as to whether HRV is altered at rest (Mercier et al., 2022). These discrepancies are likely due to the variety of physiologic monitoring methods and participant cohorts (different age groups, time since injury, etc.) evaluated in the literature (Mercier et al., 2022). At least two studies have reported between‐group differences (mTBI vs. controls) in HRV parameters at rest in the sub‐acute phase post‐mTBI (Purkayastha et al., 2019; Sung, Chen, et al., 2016). Cross‐sectional studies comparing asymptomatic individuals years following mTBI and uninjured controls have reported between‐group differences in HRV (Hilz et al., 2011, 2016). Hilz et al. recruited 20 asymptomatic participants with a history of mTBI and 20 controls (Hilz et al., 2011). During standing, the mTBI group had lower SDNN and LF compared to controls (Hilz et al., 2011). Another study by Hilz et al. had similar findings, reporting lower SDNN, RMSSD, LF, and HF in the mTBI group compared to controls at rest (Hilz et al., 2016). Both studies by Hilz et al. used ultra‐short‐term recordings and inconsistently reported methodological confounders (Mercier et al., 2022). The current study is the first, to our knowledge, to report differences in HRV between adults with months‐to‐years of PPCS presenting with exercise intolerance and age/sex‐matched controls (absent of lifetime history of TBI) in both seated and standing postures.

Many factors are known to influence HRV in addition to posture and test condition, including age (Brinth et al., 2024; Nunan et al., 2010), sex (Brinth et al., 2024; Nunan et al., 2010), menstrual cycle stage (Schmalenberger et al., 2019) and level of physical activity (Natarajan et al., 2020; Rennie et al., 2023) Despite both groups in this study (mTBI and controls) being within the normative ranges for presented HRV metrics (Brinth et al., 2024; Nunan et al., 2010), this does not suggest a lack of clinical importance of these findings. Given most participants with PPCS reported not having returned to their pre‐injury level of physical activity, this may be a primary contributor to lower HRV within the PPCS group, as there is a well‐established direct relationship between physical activity and time‐domain HRV metrics (Natarajan et al., 2020; Rennie et al., 2023) Reduction in HRV can also result from prolonged bed rest/inactivity (Ferretti et al., 2009).

The PPCS cohort appears to have been relatively active prior to their injury, with 30% participating in organized sport at the time of injury. However, at the time of study enrollment, participants reported having returned, on average, to only 28% of pre‐morbid activity (where 100% represents return to the same level/amount of exercise as prior to injury). Further, at the time of study enrollment, only 16% of participants with PPCS reported meeting physical activity guidelines. Participants with PPCS self‐reported completing a mean of 72 min (range: 0–420 min) of moderate‐to‐vigorous exercise in the week prior to the assessment, as measured using the GLTEQ, compared to the 378 min (range: 0–1890 min) reported by the control cohort. This is likely explained by the high degree of symptomatic exercise intolerance in the PPCS group, as evident on evaluation with the BCTT (PPCS participants reached a mean of 8.7 stages). This provides further evidence to suggest cardiovascular deconditioning may contribute to the lower resting HRV seen in this cohort of participants with PPCS and exercise intolerance, compared to healthy controls.

### 4.2 Effect of medication on resting HRV and BP

Sensitivity analysis was completed to explore the potential influence of medications on HR, HRV, BP, and potential contribution to between‐group differences. This cohort, as is common in participants with chronic PPCS, had a high rate of polypharmacy, and the majority of participants were taking at least one medication. Only three participants were not taking any medication. All participants were on a stable medication regimen (no change to medication(s) or dosage for ≥1 month) prior to study participation. Sensitivity analysis of groups excluding participants taking cardiovascular medications or anti‐epileptic medications largely confirmed the primary analysis findings; specifically, between‐group differences across multiple HRV parameters in the seated posture remained significant.

Results of the sensitivity analysis excluding participants taking psychiatric medications (including, anti‐depressants, anti‐psychotics or neurostimulant), revealed that between‐group differences (HR, HRV, BP) in both the seated and standing conditions were not significant. This sub‐group was less than half the size of the total cohort (*n* = 23 vs. *n* = 50). Participants excluded from this analysis were taking a variety of classes of medications, includingTCAs, tetracyclic antidepressants, SSRIs, serotonin norepinephrine reuptake inhibitors, norepinephrine dopamine reuptake inhibitor, atypical anti‐psychotic, and neurostimulants (see Table S1). As the majority of the literature on this topic has evaluated the effect of TCAs and SSRIs on HRV (Fiani et al., 2023; Kemp et al., 2016; Licht et al., 2010; Noordam et al., 2016; O'Regan et al., 2015), separate sensitivity analyses were completed excluding individuals taking TCAs or SSRIs. The results of these analyses largely mirrored the findings of the primary analysis; there were between‐group differences for multiple seated HRV and BP metrics. Therefore, we suggest that between‐group differences are unlikely being driven by medication use in the PPCS group.

The evidence for the influence of anti‐depressants on HRV outcomes is mixed (Fiani et al., 2023; Kemp et al., 2016). A meta‐analysis by Fiani et al. included pre‐post and placebo‐controlled trials evaluating the impact of anti‐depressants on autonomic function (Fiani et al., 2023). They found mixed evidence for the influence of SSRIs on HRV metrics where there was an increase in RMSSD in pre‐post studies and a decrease in RMSSD in placebo‐controlled RCTs (Fiani et al., 2023). TCAs, however, were associated with a decrease in pNN50, RMSSD, and HF (Fiani et al., 2023).

### 4.3 Change in HRV and BP from seated‐to‐standing

To further characterize autonomic cardiovascular function in PPCS, change in HRV and BP metrics from seated‐to‐standing were examined. Controls had a significantly greater increase in HR, LF norm, LF/HF, and BP (DBP, MAP) compared to participants with PPCS. Other metrics decreased significantly between postures in controls compared to participants with PPCS, including RMSSD, pNN50, HF norm, and SD1. HR and HRV response to standing changed in the expected direction in both groups where HR increase (albeit more in controls) was accompanied by an expected decrease in HRV metrics (again, to a greater degree in controls). This reflects the expected decrease in vagal tone with postural change from seated to standing (Smith et al., 1994). The greater decrease in HRV in the control group suggests improved vagal withdrawal in response to standing. DBP and MAP increased slightly in the control group between postures, while a slight decrease was observed in the PPCS group. This data may reflect altered autonomic cardiovascular function in participants with PPCS and provide a physiologic target for future interventions, including aerobic exercise (Mercier et al., 2020; Pelo et al., 2023). It is also possible the ≥1 min transition between postures was not sufficient for physiologic metrics to stabilize (especially in the PPCS group), thus accounting for some of the between‐group differences.

Hilz et al. evaluated changes in HRV metrics from supine‐to‐stand in 20 asymptomatic participants with a history of mTBI compared to controls (Hilz et al., 2011). They reported a significant decrease in RMSSD and HF from supine‐to‐standing in controls, but not in those with a history of mTBI (Hilz et al., 2011). While Hilz et al. did not report delta change between postures, our presented results are consistent with their findings; individuals had significantly less change in HR/HRV metrics in response to postural change post‐mTBI compared to controls. Given the between‐group differences revealed in this study with a modest physiologic challenge (seated‐to‐standing), evaluating HRV and BP response to greater physiologic perturbation is warranted (i.e., tilt‐table‐test, Valsalva maneuver, cold pressor test). This would allow for further characterization of autonomic function in adults with persistent symptoms in the chronic phase post‐injury.

### 4.4 Clinical implications

HRV metrics are of clinical importance as they have been associated with cardiovascular health and mortality (Hillebrand et al., 2013; Jarczok et al., 2022). Clinical cut‐offs (using SDNN metric) for cardiovascular risk stratification (Kleiger et al., 2005) and an association between HRV and risk of fatal/non‐fatal cardiovascular disease have been established (Hillebrand et al., 2013). Therefore, optimization of HRV is of clinical importance in all patient populations, including adults with PPCS. HRV may be a targetable measure in PPCS management that could be followed over time. Exercise is known to increase resting HRV (Natarajan et al., 2020; Rennie et al., 2023) and in recent years exercise has increasingly been recognized as an important rehabilitation tool in the treatment of mTBI (Leddy et al., 2023; Schneider et al., 2023).

In individuals with PPCS, many factors may limit engagement in physical activity, contributing to physical deconditioning over time. These factors include exercise intolerance, overall symptom burden, kinesiophobia, and fear avoidance, among others. Other work has demonstrated that prescription of sub‐symptom threshold aerobic exercise can both be tolerated and aid in reducing symptom burden and improving quality of life in adults with PPCS (Mercier et al., 2024, 2025). Prescription of exercise in adults with PPCS requires adequate exercise testing to assess symptom response, prescription in line with the most up‐to‐date literature, and careful monitoring. For clinicians and other health professionals, resources for the prescription of sub‐symptom threshold aerobic exercise have been published (Bezherano et al., 2021). Thus far, data demonstrate that a prolonged duration of engagement in exercise (>12 weeks) at a moderate‐to‐vigorous intensity is likely required to start seeing HRV changes with exercise in this patient population (Mercier et al., 2024).

These results are not generalizable to all adults with PPCS. These data are not representative of individuals without exercise intolerance who have been able to return to their pre‐morbid level of physical activity nor individuals who have a diagnosis of autonomic dysfunction such as POTS.

### 4.5 Strengths and limitations

There were several strengths of this study, which built on previous literature using robust methods to evaluate differences in autonomic parameters between adults with PPCS and exercise intolerance versus healthy controls. External stressors, including caffeine (Grant et al., 2023), alcohol (Brunner et al., 2021), and vigorous exercise (Burma et al., 2020) are known to influence the autonomic nervous system but are seldom reported in study methods (Mercier et al., 2022). The present study used strict pre‐participation guidelines (Mercier et al., 2022) advising participants to abstain from caffeine, alcohol, vigorous physical activity, and inhaler use for ≥6 h prior to assessment start. Several medications can also influence HR and BP. The participants with PPCS recruited for this study reflect a real‐world sample of individuals with months to years of PPCS where polypharmacy is common. Therefore, a sensitivity analysis was completed to better understand the potential effect of medications on the results. This study used 4‐min recordings, which have been shown to reliably approximate longer recordings (Burma, Graver, et al., 2021); much research in the field continues to use ultra‐short term (<3 min) recordings (Mercier et al., 2022), which are less reliable. All participants were age (±3 years) and sex‐matched, and our cohort size was larger than much of the published literature in this field (Mercier et al., 2022). Control participants were also screened for a lifetime history of diagnosed or suspected TBI. Lastly, this study fills a gap in the literature by characterizing autonomic function in adults with months to years of PPCS, where to date the vast majority of the literature has focused on adolescents with sport‐related concussion who are in the acute phase of recovery (Mercier et al., 2022).

There were several limitations to this study. Respiration rate was not measured. Especially rapid respiratory rates could impact HF norm (Shaffer & Ginsberg, 2017); although this measure was not found to be significantly different between groups. Future studies should consider using a controlled breathing protocol (Ellingson, Singh, Ellingson, Sirant, et al., 2022). Menstrual cycle stage was not controlled for, despite a known general decrease in HRV from the follicular to luteal phase (Schmalenberger et al., 2019). Due to sample heterogeneity and the number of pre‐menopausal, peri‐menopausal, and post‐menopausal females across a broad age range, there was insufficient power to explore the impact of different phases of the menstrual cycle on outcomes. This should be explored directly in future studies. There was a greater number of female (74%) versus male participants. This is unsurprising, given females are more likely to have a prolonged recovery and are also more likely to seek specialized medical care (Putukian et al., 2023). Including participants from 3 months to 5 years post‐mTBI may have introduced heterogeneity in the degree of deconditioning. Data on the exact duration of adjunct therapies (occupation/physical/vestibular therapy) that were being undergone concurrently or had been engaged in prior to study participation were not collected. Physical activity levels were self‐reported in both cohorts, and unsurprisingly, significantly more controls reported meeting physical activity guidelines than participants with PPCS in the week prior to data collection. Given their high degree of exercise intolerance, most of the PPCS cohort had been largely sedentary since their injury. Actigraphy could have been used to further characterize baseline physical activity in both groups. While the PPCS group did show a significant difference in autonomic measures with postural change compared to controls, a greater physiologic challenge (i.e., tilt‐table‐test, Valsalva maneuver, cold pressor test) would be useful to further probe ANS function in this patient cohort. Finally, a specific questionnaire measure of autonomic symptoms could have been included.

## 5 CONCLUSION

This study evaluated autonomic cardiovascular function in adults with PPCS and exercise intolerance. Higher resting HR and BP in participants with PPCS and exercise intolerance may reflect deconditioning following injury, with clinical implications for cardiovascular health. Controls had significantly greater changes in HR, BP, and HRV parameters from seated to standing compared to participants with PPCS, which may suggest altered autonomic cardiovascular function post‐injury in this cohort. Exercise is known to alter resting HRV and is increasingly being recognized for its important role in mTBI rehabilitation (Leddy et al., 2023; Schneider et al., 2023). This study provides evidence of altered autonomic cardiovascular function in participants with PPCS compared to age/sex‐matched controls, providing emerging evidence to suggest these physiological metrics could be monitored during rehabilitation.

## FUNDING INFORMATION

This trial was supported by a New Frontiers in Research Fund Exploration Grant, Foundation of Physical Medicine and Rehabilitation Mid‐Career Grant, and a Hotchkiss Brain Institute PFUND Award. Additional support was provided from the Hotchkiss Brain Institute. LJM was supported by a Graduate Studentship in Patient‐Oriented Research from the Alberta SPOR Support Unit (jointly funded by Alberta Innovates‐Health Solutions and the Canadian Institute of Health Research), a Dr. Matthew Galati Brain Changer Award from Brain Canada, a Graduate Studentship from the Integrated Concussion Research Program, and University of Calgary Scholarships. The Dr. Matthew Galati Brain Changer Award has been made possible by the Canada Brain Research Fund (CBRF), an innovative arrangement between the Government of Canada (through Health Canada) and Brain Canada Foundation, and by the Brain Changes Initiative. The views expressed herein do not necessarily represent the views of the Minister of Health or the Government of Canada. SJM was supported by an O'Brien Centre Summer Studentship Award and University of Calgary Scholarships. JSB was supported by funding from the University of Calgary (John D Petrie QC Memorial Scholarship and Brain Create) and the Natural Sciences and Engineering Research Council (Alexander Graham Bell Canada Graduate Scholarship‐Doctoral Program). JB was supported by an Integrated Concussion Research Program Summer Studentship. JMJ was supported by an Alberta Graduate Excellence Scholarship. JDS holds a NSERC Discovery grant, as well as funding from Brain Canada and the Branch Out Neurological Society. ADH holds a Canada Research Chair in Magnetic Resonance Spectroscopy in Brain Injury and is supported by the Hotchkiss Brain Institute, Alberta Children's Hospital Research Institute, NSERC, and CIHR.

## CONFLICT OF INTEREST STATEMENT

S. P. Dukelow has received consulting fees from AbbVie, Merz, and Ipsen for work unrelated to this manuscript.

## ETHICS STATEMENT

Ethics approval was received by the University of Calgary Conjoint Health Research Ethics Board (REB18‐1329).

## Supporting information


Appendix S1.

## Data Availability

Available on reasonable request.
