# Supplementary material for: Altered autonomic cardiovascular function in adults with persisting post‐concussive symptoms and exercise intolerance
Source: Physiol Rep. 2025 Jun 11;13(11):e70378. doi: 10.14814/phy2.70378 (PMC12159307; doi:10.14814/phy2.70378)
Supplement: Supplementary file 1 — Appendix S1. [file PHY2-13-e70378-s001.docx]

**Altered Autonomic Cardiovascular Function in Adults with Persisting Post-Concussive Symptoms and Exercise Intolerance**

Supplementary Materials

| Supplementary Figure 1 | Resting seated and standing HRV and BP: PPCS vs age/sex-matched controls |
| --- | --- |
| Supplementary Table 1 | PPCS Participant medications |
| Supplementary Table 2 | Sensitivity Analysis excluding PPCS participants on cardiac medications: Resting seated and standing HRV and BP in PPCS vs age/sex-matched controls |
| Supplementary Table 3 | Sensitivity Analysis excluding PPCS participants on pain or anti-epileptic medications: Resting seated and standing HRV and BP in PPCS vs age/sex-matched controls |
| Supplementary Table 4 | Sensitivity Analysis excluding PPCS participants on anti-depressant/anti-psychotic/neurostimulant medications: Resting seated and standing HRV and BP in PPCS vs age/sex-matched controls |
| Supplementary Table 5 | Sensitivity Analysis excluding PPCS participants on tricyclic anti-depressant (TCA) medications: Resting seated and standing HRV and BP in PPCS vs age/sex-matched controls |
| Supplementary Table 6 | Sensitivity Analysis excluding PPCS participants on selective serotonin reuptake inhibitor (SSRI) medications: Resting seated and standing HRV and BP in PPCS vs age/sex-matched controls |

Supplementary Figure 1 Resting seated and standing HRV and BP: PPCS vs age/sex-matched controls

Groups are participants with PPCS (n=50) and age/sex-matched controls (n=50). Mean and 95% confidence intervals from models of resting values for seated and standing postures are presented for each group. Outcomes are **(A)** HR; **(B)** pNN50; **(C)** RMSSD; **(D)** SDNN; **(E)** LF norm; **(F)** HF norm; **(G)** LF/HF ratio; **(H)** SD1; **(I)** SD2; **(J)** SBP; **(K)** DBP; **(L)** LF gain.

**A B C D**


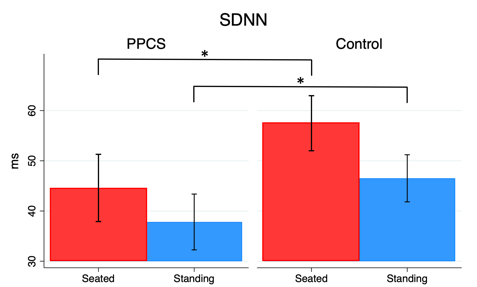

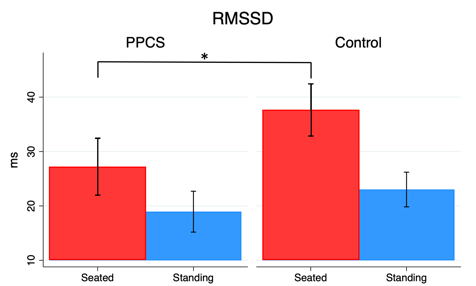

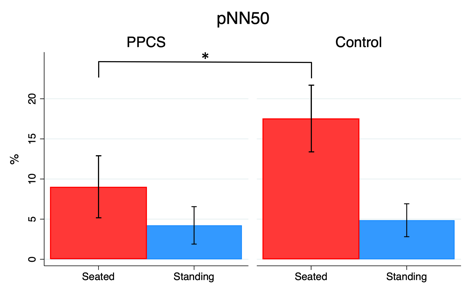

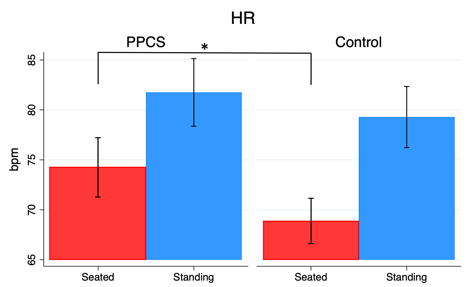


**E F G H**


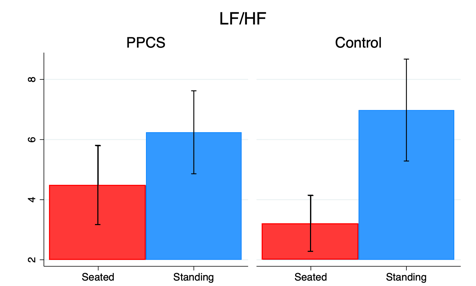

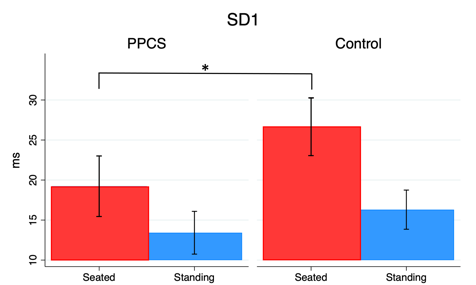

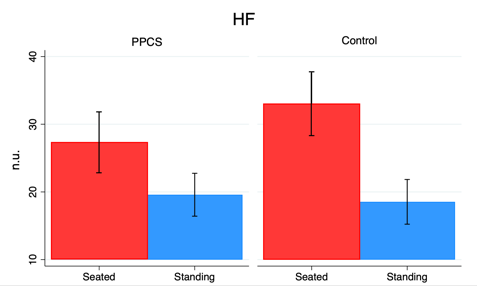

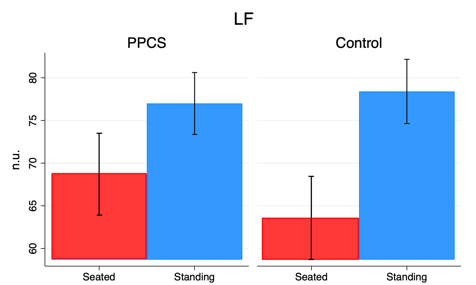


**I J K L**


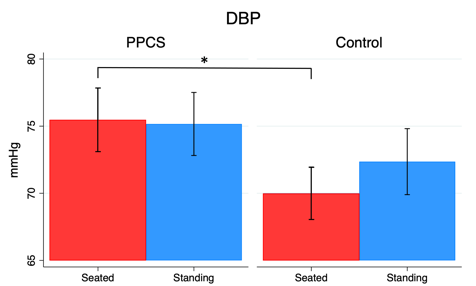

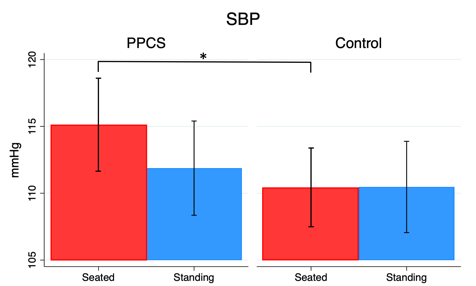

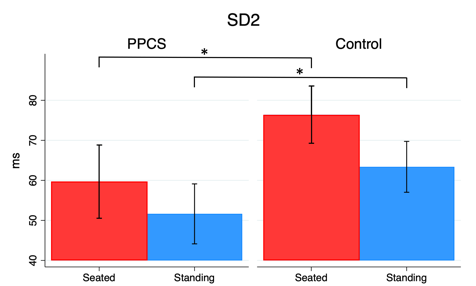


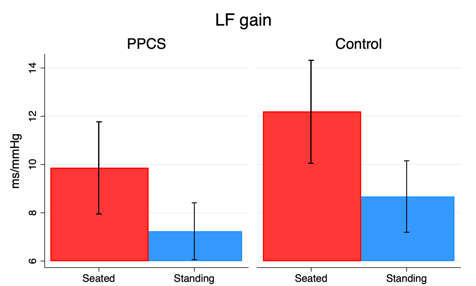


Supplementary Table 1 PPCS participant medications

|  | PPCS participants  (n=50) |
| --- | --- |
| **Anti-depressant/Anti-psychotic/Neurostimulant, (n [≥1 of the below], %)** | 27 (54) |
| Amitriptyline (Elavil) | 8 (16) |
| Aripiprazole (Abilify) | 1 (2) |
| Bupropion (Wellbutrin) | 1 (2) |
| Buspirone (Buspar) | 1 (2) |
| Citalopram (Celexa) | 2 (4) |
| Clomipramine (Anafranil) | 1 (2) |
| Desvenlafaxine (Pristiq) | 1 (2) |
| Duloxetine (Cymbalta) | 2 (4) |
| Escitalopram (Cipralex) | 2 (4) |
| Fluoxetine (Prozac) | 1 (2) |
| Lisdexamfetamine (Vyvanse) | 4 (8) |
| Methylphenidate (Concerta/Ritalin) | 2 (4) |
| Mirtazapine (Remeron) | 1 (2) |
| Nortriptyline (Pamelor) | 1 (2) |
| Quetiapine (Seroquel) | 1 (2) |
| Sertraline (Zoloft) | 3 (6) |
| Venlafaxine (Effexor) | 3 (6) |
| Vilazodone (Viibryd) | 1 (2) |
| Vortioxetine (Trintellix/Brintellix) | 1 (2) |
| **Anti-epileptics, n (%)** | 4 (8) |
| Gabapentin (Neurontin) | 1 (2) |
| Pregabalin (Lyrica) | 3 (6) |
| **Anti-nausea/GI, n (%)** | 8 (16) |
| Dexlansoprazole (Dexilant) | 1 (2) |
| Esomeprazole (Nexium) | 1 (2) |
| Metaclopromide (Primperan/Reglan) | 2 (4) |
| Ondansetron (Zofran) | 1 (2) |
| Pantoprazole (Protonix/Pantoloc) | 3 (6) |
| Prucalopride (Resotran/Resolor/Motegrity) | 1 (2) |
| **Cardiovascular, n (%)** | 3 (6) |
| Candesartan (Atacand) | 1 (2) |
| Hydrochlorothiazide (Apo-hydro) | 2 (4) |
| Perindopril (Coversyl/Coversum/Aceon) | 1 (2) |
| **Endocrine, n (%)** | 7 (14) |
| Desiccated thyroid | 1 (2) |
| Levothyroxine (Synthroid) | 2 (4) |
| Somatropin (Genotropin/Humatrope) | 4 (8) |
| **Headache, n (%)** | 10 (20) |
| Almotriptan (Axert) | 1 (2) |
| Botox | 6 (12) |
| Erenumab (Aimovig) | 1 (2) |
| Galcanezumab (Emgality) | 1 (2) |
| Rizatriptan (Maxalt) | 3 (6) |
| **Hormone replacement therapy, n (%)** | 2 (4) |
| Estriol/Estradiol Cream or insert (Biest/Vagifem) | 2 (4) |
| Oral progesterone (Prometrium) | 1 (2) |
|  |  |
|  | PPCS participants  (n=50) |
| **Metabolic, n (%)** | 5 (10) |
| Dulaglutide (Trulicity) | 1 (2) |
| Empagliflozin (Jardiance) | 1 (2) |
| Ezetimibe (Ezetrol) | 2 (4) |
| Metformin (Glucophage) | 1 (2) |
| Prevastatin (Pravachol) | 1 (2) |
| Rosuvastatin (Crestor) | 3 (6) |
| Semaglutide (Ozempic) | 1 (2) |
| **Pain, n (%)** | 14 (28) |
| Baclofen (Lioresal) | 1 (2) |
| Cannabidiol (CBD) | 3 (6) |
| Celecoxib (Celebrex) | 1 (2) |
| Cyclobenzaprine, oral | 2 (4) |
| Cyclobenzaprine, cream | 2 (4) |
| Diclofenac, oral (Cambia) | 5 (10) |
| Diclofenac, cream | 1 (2) |
| Naproxen (Aleve) | 4 (8) |
| **Respiratory/Antihistamine, n (%)** | 4 (8) |
| Azelastine Hydrochloride & Fluticasone Propionate nasal spray (Dymista) | 1 (2) |
| Albuterol (Ventolin) | 1 (2) |
| Budesonide/formoterol (Symbicort) | 1 (2) |
| Bilastine (Blexten) | 1 (2) |
| Cetirizine (Zyrtec) | 1 (2) |
| Montelukast (Singular) | 1 (2) |
| Rupatadine (Rupall) | 1 (2) |
| **Sleep, n (%)** | 15 (30) |
| Melatonin | 9 (18) |
| Lemborexant (Dayvigo) | 1 (2) |
| Tryptophan | 3 (6) |
| Zopiclone (Imovane) | 3 (6) |

Supplementary Table 2 Sensitivity Analysis excluding PPCS participants on cardiac medications: Resting seated and standing HRV and BP in PPCS vs age/sex-matched controls

Mean difference and 95% confidence intervals from the random intercept model of resting seated and standing HRV and BP between participants with PPCS and controls. All participants with PPCS taking cardiovascular medications were excluded from this analysis.

|  | Seated Comparison | | | | | Standing Comparison | | | | |
| --- | --- | --- | --- | --- | --- | --- | --- | --- | --- | --- |
|  | PPCS  (n=46) | Controls  (n=46) |  |  |  | PPCS  (n=46) | Controls  (n=46) |  |  |  |
|  | Mean (SE) | Mean (SE) | Mean difference | 95% CI | p value | Mean (SE) | Mean (SE) | Mean difference | 95% CI | p value |
| HR (bpm) | 73.9 (1.6) | 68.9 (1.2) | 5.003 | (1.354, 8.652) | 0.007* | 81.6 (1.9) | 79.5 (1.7) | 2.055 | (-2.282, 6.391) | 0.353 |
| pNN50 (%) | 9.5 (2.1) | 17.1 (2.3) | -7.594 | (-13.479, -1.710) | 0.011* | 4.5 (1.3) | 4.5 (1.1) | -0.041 | (-3.249, 3.167) | 0.980 |
| RMSSD (ms) | 27.8 (2.9) | 37.2 (2.6) | -9.370 | (-16.022, -2.718) | 0.006* | 19.5 (2.0) | 22.4 (1.8) | -2.970 | (-8.049, 2.108) | 0.252 |
| SDNN (ms) | 44.7 (3.7) | 56.8 (3.0) | -12.121 | (-19.952, -4.291) | 0.002* | 38.7 (3.0) | 44.8 (2.2) | -6.152 | (-12.579, 0.276) | 0.061 |
| LF norm (n.u.) | 68.0 (2.7) | 63.5 (2.6) | 4.555 | (-2.580, 11.691) | 0.211 | 76.6 (1.9) | 79.0 (1.9) | -2.439 | (-8.205, 3.328) | 0.407 |
| HF norm (n.u.) | 27.9 (2.4) | 33.1 (2.5) | -5.165 | (-12.074, 1.745) | 0.143 | 20.0 (1.7) | 17.8 (1.6) | 2.145 | (-2.756, 7.045) | 0.391 |
| LF/HF | 4.4 (0.7) | 3.2 (0.5) | 1.135 | (-0.317, 2.588) | 0.126 | 6.0 (0.7) | 7.2 (0.9) | -1.159 | (-3.545, 1.226) | 0.341 |
| SD1 (ms) | 19.7 (2.0) | 26.4 (1.9) | -6.639 | (-11.348, -1.930) | 0.006* | 13.8 (1.4) | 15.9 (1.3) | -2.103 | (-5.769, 1.563) | 0.261 |
| SD2 (ms) | 59.8 (4.9) | 75.6 (4.0) | -15.808 | (-26.293, -5.323) | 0.003* | 52.8 (4.0) | 61.0 (3.0) | -8.232 | (-17.527, 1.064) | 0.083 |
| SBP^a^ (mmHg) | 114.3 (1.7) | 110.5 (1.6) | 3.826 | (-0.272, 7.924) | 0.067 | 111.6 (1.7) | 110.4 (1.9) | 1.239 | (-3.400, 5.879) | 0.601 |
| DBP^a^ (mmHg) | 75.2 (1.3) | 70.1 (1.1) | 5.065 | (1.863, 8.267) | 0.002* | 74.9 (1.2) | 72.5 (1.3) | 2.446 | (-0.900, 5.791) | 0.152 |
| MAP^a^ (mmHg) | 88.2 (1.3) | 83.6 (1.1) | 4.652 | (1.540, 7.764) | 0.003* | 87.1 (1.3) | 85.1 (1.4) | 2.043 | (-1.374, 5.461) | 0.241 |

Abbreviations: BP = blood pressure; DBP = diastolic blood pressure; HR = heart rate; HRV = heart rate variability; HF norm = high frequency power in normalized units; LF gain = low frequency gain; LF norm = low frequency power in normalized units; LF/HF ratio = low frequency to high frequency ratio; MAP = mean arterial pressure; pNN50 = percent of RR intervals differing by more than 50 ms; PPCS = persistent post-concussive symptoms; RMSSD = root mean squared of successive RR interval differences; SBP = systolic blood pressure; SDNN = standard deviation of RR intervals; SD1 = Pointcaré plot standard deviation perpendicular the line of identity; SD2 = Pointcaré plot standard deviation along the line of identity

* statistically significant at p<0.05

^a^ average of two manual brachial BPs

Supplementary Table 3 Sensitivity Analysis excluding PPCS participants on pain or anti-epileptic medications: Resting seated and standing HRV and BP in PPCS vs age/sex-matched controls

Mean difference and 95% confidence intervals from the random intercept model of resting seated and standing HRV and BP between participants with PPCS and controls. All participants with PPCS taking pain/anti-epileptic medications were excluded from this analysis.

|  | Seated Comparison | | | | | Standing Comparison | | | | |
| --- | --- | --- | --- | --- | --- | --- | --- | --- | --- | --- |
|  | PPCS  (n=32) | Controls  (n=32) |  |  |  | PPCS  (n=32) | Controls  (n=32) |  |  |  |
|  | Mean (SE) | Mean (SE) | Mean difference | 95% CI | p value | Mean (SE) | Mean (SE) | Mean difference | 95% CI | p value |
| HR (bpm) | 74.6 (2.1) | 68.17 (1.5) | 6.472 | (1.699, 11.245) | 0.008* | 78.8 (2.1) | 82.2 (2.5) | 3.416 | (-1.737, 8.569) | 0.194 |
| pNN50 (%) | 8.7 (2.1) | 19.1 (2.9) | -10.394 | (-17.558, -3.229) | 0.004* | 4.0 (1.3) | 5.6 (1.5) | -1.629 | (-5.016, 1.758) | 0.346 |
| RMSSD (ms) | 27.5 (3.2) | 40.0 (3.4) | -12.506 | (-20.365, -4.647) | 0.002* | 19.5 (2.2) | 24.1 (2.4) | -4.589 | (-10.748, 1.569) | 0.144 |
| SDNN (ms) | 47.5 (4.9) | 59.2 (3.8) | -11.786 | (-22.805, -0.767) | 0.036* | 39.8 (3.3) | 47.3 (3.4) | -7.419 | (-16.607, 1.768) | 0.113 |
| LF norm (n.u.) | 69.2 (3.4) | 62.8 (3.3) | 6.369 | (-1.951, 14.689) | 0.134 | 76.0 (2.3) | 76.7 (2.4) | -0.741 | (-8.430, 6.948) | 0.850 |
| HF norm (n.u.) | 26.5 (2.9) | 33.9 (3.1) | -7.404 | (-15.082, 0.274) | 0.059 | 20.3 (2.0) | 20.0 (2.1) | 0.438 | (-5.750, 6.626) | 0.890 |
| LF/HF | 5.0 (1.0) | 3.2 (0.7) | 1.763 | (-0.174, 3.699) | 0.074 | 6.0 (0.9) | 6.5 (1.1) | -0.562 | (-3.421, 2.297) | 0.700 |
| SD1 (ms) | 19.5 (2.3) | 28.3 (2.4) | -8.862 | (-14.412, -3.312) | 0.002* | 13.8 (1.6) | 17.1 (1.7) | -3.250 | (-7.640, 1.139) | 0.147 |
| SD2 (ms) | 64.0 (6.5) | 78.5 (4.9) | -14.476 | (-29.844, 0.892) | 0.065 | 54.5 (4.4) | 64.3 (4.6) | -9.795 | (-22.096, 2.506) | 0.119 |
| SBP^a^ (mmHg) | 117.9 (2.3) | 112.1 (1.9) | 5.781 | (-0.238, 11.801) | 0.060 | 113.4 (2.1) | 112.7 (2.3) | 0.781 | (-5.268, 6.830) | 0.800 |
| DBP^a^ (mmHg) | 75.9 (1.5) | 70.9 (1.2) | 5.000 | (1.233, 8.767) | 0.009* | 75.9 (1.5) | 74.1 (1.7) | 1.797 | (-2.251, 5.845) | 0.384 |
| MAP^a^ (mmHg) | 89.9 (1.6) | 84.7 (1.3) | 5.260 | (1.138, 9.383) | 0.012* | 88.4 (1.5) | 87.0 (1.7) | 1.458 | (-2.855, 5.772) | 0.508 |

Abbreviations: BP = blood pressure; DBP = diastolic blood pressure; HR = heart rate; HRV = heart rate variability; HF norm = high frequency power in normalized units; LF gain = low frequency gain; LF norm = low frequency power in normalized units; LF/HF ratio = low frequency to high frequency ratio; MAP = mean arterial pressure; pNN50 = percent of RR intervals differing by more than 50 ms; PPCS = persistent post-concussive symptoms; RMSSD = root mean squared of successive RR interval differences; SBP = systolic blood pressure; SDNN = standard deviation of RR intervals; SD1 = Pointcaré plot standard deviation perpendicular the line of identity; SD2 = Pointcaré plot standard deviation along the line of identity

* statistically significant at p<0.05

^a^ average of two manual brachial BPs

Supplementary Table 4 Sensitivity Analysis excluding PPCS participants on anti-depressant/anti-psychotic/neurostimulant medications: Resting seated and standing HRV and BP in PPCS vs age/sex-matched controls

Mean difference and 95% confidence intervals from the random intercept model of resting seated and standing HRV and BP between participants with PPCS and controls. All participants with PPCS taking anti-depressant/anti-psychotic/neurostimulant medications were excluded from this analysis.

|  | Seated Comparison | | | | | Standing Comparison | | | | |
| --- | --- | --- | --- | --- | --- | --- | --- | --- | --- | --- |
|  | PPCS  (n=22) | Controls  (n=22) |  |  |  | PPCS  (n=23) | Controls  (n=23) |  |  |  |
|  | Mean (SE) | Mean (SE) | Mean difference | 95% CI | p value | Mean (SE) | Mean (SE) | Mean difference | 95% CI | p value |
| HR (bpm) | 70.1 (1.7) | 68.7 (1.8) | 1.434 | (-3.305, 6.172) | 0.553 | 77.7 (2.0) | 78.1 (2.5) | -0.397 | (-6.649, 5.856) | 0.901 |
| pNN50 (%) | 14.3 (3.6) | 17.0 (3.9) | -2.740 | (-12.773, 7.292) | 0.592 | 6.4 (2.2) | 4.5 (1.8) | 1.876 | (-3.204, 6.957) | 0.469 |
| RMSSD (ms) | 35.4 (4.6) | 37.6 (4.7) | -2.234 | (-13.165, 8.697) | 0.689 | 23.7 (3.1) | 21.7 (2.5) | 1.982 | (-5.440, 9.403) | 0.601 |
| SDNN (ms) | 52.0 (5.8) | 58.9 (5.0) | -6.887 | (-21.035, 7.260) | 0.340 | 45.0 (4.4) | 45.1 (3.7) | -0.126 | (-11.235, 10.982) | 0.982 |
| LF norm (n.u.) | 63.1 (4.6) | 67.0 (3.5) | -3.924 | (-13.235, 5.387) | 0.409 | 76.5 (2.7) | 80.0 (2.7) | -3.550 | (-11.098, 3.998) | 0.357 |
| HF norm (n.u.) | 32.3 (4.3) | 30.2 (3.4) | 2.123 | (-8.093, 12.338) | 0.684 | 19.7 (2.4) | 17.3 (2.4) | 2.390 | (-4.281, 9.061) | 0.483 |
| LF/HF | 4.6 (1.3) | 3.9 (0.9) | 0.693 | (-1.896, 3.282) | 0.600 | 6.1 (0.9) | 7.9 (1.5) | -1.830 | (-5.169, 1.509) | 0.283 |
| SD1 (ms) | 25.1 (3.3) | 26.6 (3.4) | -1.583 | (-9.159, 5.993) | 0.682 | 16.8 (2.2) | 15.4 (1.8) | 1.406 | (-3.985, 6.796) | 0.609 |
| SD2 (ms) | 68.8 (7.6) | 78.5 (6.5) | -9.656 | (-28.312, 9.000) | 0.310 | 61.3 (5.9) | 61.6 (5.0) | -0.310 | (-15.602, 14.982) | 0.968 |
| SBP^a^ (mmHg) | 108.6 (2.2) | 111.7 (2.2) | -3.136 | (-8.137, 1.864) | 0.219 | 106.2 (2.1) | 110.4 (2.7) | -4.217 | (-10.339, 1.904) | 0.177 |
| DBP^a^ (mmHg) | 70.7 (1.6) | 70.5 (1.3) | 0.227 | (-3.575, 4.029) | 0.907 | 70.9 (1.7) | 72.1 (1.9) | -1.304 | (-6.245, 3.636) | 0.605 |
| MAP^a^ (mmHg) | 83.3 (1.6) | 84.2 (1.6) | -0.894 | (-4.912, 3.125) | 0.663 | 82.6 (1.6) | 84.9 (1.9) | -2.275 | (-7.038, 2.487) | 0.349 |

Abbreviations: BP = blood pressure; DBP = diastolic blood pressure; HR = heart rate; HRV = heart rate variability; HF norm = high frequency power in normalized units; LF gain = low frequency gain; LF norm = low frequency power in normalized units; LF/HF ratio = low frequency to high frequency ratio; MAP = mean arterial pressure; pNN50 = percent of RR intervals differing by more than 50 ms; PPCS = persistent post-concussive symptoms; RMSSD = root mean squared of successive RR interval differences; SBP = systolic blood pressure; SDNN = standard deviation of RR intervals; SD1 = Pointcaré plot standard deviation perpendicular the line of identity; SD2 = Pointcaré plot standard deviation along the line of identity

* statistically significant at p<0.05

^a^ average of two manual brachial BPs

Supplementary Table 5 Sensitivity Analysis excluding PPCS participants on tricyclic anti-depressant (TCA) medications: Resting seated and standing HRV and BP in PPCS vs age/sex-matched controls

Mean difference and 95% confidence intervals from the random intercept model of resting seated and standing HRV and BP between participants with PPCS and controls. All participants with PPCS taking anti-depressant/anti-psychotic/neurostimulant medications were excluded from this analysis.

|  | Seated Comparison | | | | | Standing Comparison | | | | |
| --- | --- | --- | --- | --- | --- | --- | --- | --- | --- | --- |
|  | PPCS  (n=39) | Controls  (n=39) |  |  |  | PPCS  (n=40) | Controls  (n=40) |  |  |  |
|  | Mean (SE) | Mean (SE) | Mean difference | 95% CI | p value | Mean (SE) | Mean (SE) | Mean difference | 95% CI | p value |
| HR (bpm) | 73.3 (1.6) | 69.4 (1.3) | 3.916 | (0.054, 7.778) | 0.047* | 81.1 (1.9) | 79.7 (1.8) | 1.475 | (-3.024, 5.974) | 0.520 |
| pNN50 (%) | 9.0 (2.3) | 16.8 (2.5) | -7.774 | (-14.387, -1.160) | 0.021* | 4.1 (1.3) | 4.7 (1.1) | -0.573 | (-3.941, 2.794) | 0.739 |
| RMSSD (ms) | 27.5 (3.1) | 37.1 (2.9) | -9.530 | (-16.439, -2.621) | 0.007* | 18.9 (2.1) | 22.8 (2.0) | -3.902 | (-9.504, 1.699) | 0.172 |
| SDNN (ms) | 44.7 (3.9) | 57.3 (3.3) | -12.593 | (-21.216, -3.971) | 0.004* | 37.6 (3.2) | 46.6 (2.8) | -9.010 | (-17.314, -0.705) | 0.033* |
| LF norm (n.u.) | 68.0 (2.9) | 65.6 (2.6) | 2.358 | (-4.860, 9.575) | 0.522 | 76.0 (2.0) | 79.9 (2.0) | -3.839 | (-9.864, 2.186) | 0.212 |
| HF norm (n.u.) | 28.0 (2.7) | 31.4 (2.6) | -3.364 | (-10.613, 3.885) | 0.363 | 20.2 (1.8) | 17.2 (1.8) | 2.970 | (-2.147, 8.088) | 0.255 |
| LF/HF | 4.4 (0.8) | 3.5 (0.6) | 0.907 | (-0.699, 2.512) | 0.268 | 5.9 (0.7) | 7.4 (1.0) | -1.468 | (-3.831, 0.894) | 0.223 |
| SD1 (ms) | 19.5 (2.2) | 26.3 (2.1) | -6.752 | (-12.013, -1.491) | 0.012* | 13.4 (1.5) | 16.1 (1.4) | -2.763 | (-6.874, 1.348) | 0.188 |
| SD2 (ms) | 59.8 (5.2) | 76.3 (4.4) | -16.523 | (-28.122, -4.924) | 0.005* | 51.3 (4.3) | 63.4 (3.8) | -12.186 | (-23.022, -1.350) | 0.028* |
| SBP^a^ (mmHg) | 114.2 (2.2) | 111.0 (1.8) | 3.179 | (-1.936, 8.295) | 0.223 | 111.2 (2.1) | 111.0 (2.1) | 0.175 | (-5.319, 5.669) | 0.950 |
| DBP^a^ (mmHg) | 75.4 (1.5) | 69.4 (1.1) | 5.974 | (2.384, 9.565) | 0.001* | 74.8 (1.4) | 72.1 (1.5) | 2.750 | (-1.121, 6.621) | 0.164 |
| MAP^a^ (mmHg) | 88.3 (1.6) | 83.3 (1.2) | 5.043 | (1.140, 8.945) | 0.011* | 87.0 (1.5) | 85.1 (1.5) | 1.892 | (-1.887, 5.670) | 0.326 |

Abbreviations: BP = blood pressure; DBP = diastolic blood pressure; HR = heart rate; HRV = heart rate variability; HF norm = high frequency power in normalized units; LF gain = low frequency gain; LF norm = low frequency power in normalized units; LF/HF ratio = low frequency to high frequency ratio; MAP = mean arterial pressure; pNN50 = percent of RR intervals differing by more than 50 ms; PPCS = persistent post-concussive symptoms; RMSSD = root mean squared of successive RR interval differences; SBP = systolic blood pressure; SDNN = standard deviation of RR intervals; SD1 = Pointcaré plot standard deviation perpendicular the line of identity; SD2 = Pointcaré plot standard deviation along the line of identity

* statistically significant at p<0.05

^a^ average of two manual brachial BPs

Supplementary Table 6 Sensitivity Analysis excluding PPCS participants on selective serotonin reuptake inhibitor (SSRI) medications: Resting seated and standing HRV and BP in PPCS vs age/sex-matched controls

Mean difference and 95% confidence intervals from the random intercept model of resting seated and standing HRV and BP between participants with PPCS and controls. All participants with PPCS taking anti-depressant/anti-psychotic/neurostimulant medications were excluded from this analysis.

|  | Seated Comparison | | | | | Standing Comparison | | | | |
| --- | --- | --- | --- | --- | --- | --- | --- | --- | --- | --- |
|  | PPCS  (n=40) | Controls  (n=40) |  |  |  | PPCS  (n=41) | Controls  (n=41) |  |  |  |
|  | Mean (SE) | Mean (SE) | Mean difference | 95% CI | p value | Mean (SE) | Mean (SE) | Mean difference | 95% CI | p value |
| HR (bpm) | 73.4 (1.5) | 69.5 (1.4) | 3.904 | (0.018, 7.790) | 0.049* | 81.6 (1.9) | 79.6 (1.8) | 1.989 | (-2.679, 6.658) | 0.404 |
| pNN50 (%) | 10.6 (2.4) | 18.7 (2.5) | -8.051 | (-14.896, -1.206) | 0.021* | 4.9 (1.5) | 5.0 (1.2) | -0.073 | (-3.629, 3.483) | 0.968 |
| RMSSD (ms) | 29.8 (3.1) | 38.6 (2.9) | -8.775 | (-16.425, -1.124) | 0.025* | 20.2 (2.2) | 22.9 (1.8) | -2.764 | (-8.220, 2.693) | 0.321 |
| SDNN (ms) | 47.9 (4.0) | 59.9 (3.3) | -11.937 | (-20.697, -3.178) | 0.008* | 40.1 (3.3) | 47.6 (2.8) | -7.446 | (-15.871, 0.977) | 0.083 |
| LF norm (n.u.) | 69.4 (3.0) | 63.7 (2.9) | 5.720 | (-1.825, 13.265) | 0.137 | 78.7 (1.8) | 78.1 (2.1) | 0.652 | (-4.935, 6.238) | 0.819 |
| HF norm (n.u.) | 26.9 (2.7) | 33.2 (2.7) | -6.300 | (-13.347, 0.748) | 0.080 | 18.0 (1.6) | 18.7 (1.8) | -0.615 | (-5.469, 4.239) | 0.804 |
| LF/HF | 4.9 (0.8) | 3.3 (0.6) | 1.574 | (-0.136, 3.284) | 0.071 | 6.7 (0.8) | 7.0 (1.0) | -0.260 | (-2.853, 2.334) | 0.844 |
| SD1 (ms) | 21.1 (2.2) | 27.4 (2.1) | -6.217 | (-11.573, -0.861) | 0.023* | 14.3 (1.6) | 16.2 (1.3) | -1.956 | (-5.957, 2.045) | 0.338 |
| SD2 (ms) | 64.1 (5.3) | 79.8 (4.4) | -15.706 | (-28.211, -3.200) | 0.014* | 54.8 (4.4) | 64.9 (3.8) | -10.162 | (-20.728, 0.403) | 0.059 |
| SBP^a^ (mmHg) | 114.7 (2.1) | 110.7 (1.8) | 3.975 | (-0.978, 8.928) | 0.116 | 111.9 (2.1) | 111.0 (2.0) | 0.878 | (-4.061, 5.818) | 0.728 |
| DBP^a^ (mmHg) | 74.8 (1.3) | 70.9 (1.1) | 3.825 | (0.562, 7.088) | 0.022* | 75.0 (1.3) | 73.5 (1.4) | 1.524 | (-2.004, 5.053) | 0.397 |
| MAP^a^ (mmHg) | 88.1 (1.5) | 84.2 (1.2) | 3.875 | (0.558, 7.192) | 0.022* | 87.3 (1.4) | 86.0 (1.5) | 1.309 | (-2.259, 4.877) | 0.472 |

Abbreviations: BP = blood pressure; DBP = diastolic blood pressure; HR = heart rate; HRV = heart rate variability; HF norm = high frequency power in normalized units; LF gain = low frequency gain; LF norm = low frequency power in normalized units; LF/HF ratio = low frequency to high frequency ratio; MAP = mean arterial pressure; pNN50 = percent of RR intervals differing by more than 50 ms; PPCS = persistent post-concussive symptoms; RMSSD = root mean squared of successive RR interval differences; SBP = systolic blood pressure; SDNN = standard deviation of RR intervals; SD1 = Pointcaré plot standard deviation perpendicular the line of identity; SD2 = Pointcaré plot standard deviation along the line of identity

* statistically significant at p<0.05

^a^ average of two manual brachial BPs
